# Left Ventricular Ejection Time Measured by Echocardiography Differentiates Neurobehavioral Resilience and Vulnerability to Sleep Loss and Stress

**DOI:** 10.3389/fphys.2021.795321

**Published:** 2022-01-11

**Authors:** Erika M. Yamazaki, Kathleen M. Rosendahl-Garcia, Courtney E. Casale, Laura E. MacMullen, Adrian J. Ecker, James N. Kirkpatrick, Namni Goel

**Affiliations:** ^1^Biological Rhythms Research Laboratory, Department of Psychiatry and Behavioral Sciences, Rush University Medical Center, Chicago, IL, United States; ^2^Siemens Healthineers, Inc., Mountain View, CA, United States; ^3^Division of Sleep and Chronobiology, Department of Psychiatry, Perelman School of Medicine, University of Pennsylvania, Philadelphia, PA, United States; ^4^Division of Cardiology, Department of Medicine, University of Washington, Seattle, WA, United States

**Keywords:** hemodynamics, sleep deprivation, psychological stress, neurobehavioral performance, Karolinska Sleepiness Scale, biomarkers, echocardiography, Psychomotor Vigilance Test

## Abstract

There are substantial individual differences (resilience and vulnerability) in performance resulting from sleep loss and psychosocial stress, but predictive potential biomarkers remain elusive. Similarly, marked changes in the cardiovascular system from sleep loss and stress include an increased risk for cardiovascular disease. It remains unknown whether key hemodynamic markers, including left ventricular ejection time (LVET), stroke volume (SV), heart rate (HR), cardiac index (CI), blood pressure (BP), and systemic vascular resistance index (SVRI), differ in resilient vs. vulnerable individuals and predict differential performance resilience with sleep loss and stress. We investigated for the first time whether the combination of total sleep deprivation (TSD) and psychological stress affected a comprehensive set of hemodynamic measures in healthy adults, and whether these measures differentiated neurobehavioral performance in resilient and vulnerable individuals. Thirty-two healthy adults (ages 27–53; 14 females) participated in a 5-day experiment in the Human Exploration Research Analog (HERA), a high-fidelity National Aeronautics and Space Administration (NASA) space analog isolation facility, consisting of two baseline nights, 39 h TSD, and two recovery nights. A modified Trier Social Stress Test induced psychological stress during TSD. Cardiovascular measure collection [SV, HR, CI, LVET, BP, and SVRI] and neurobehavioral performance testing (including a behavioral attention task and a rating of subjective sleepiness) occurred at six and 11 timepoints, respectively. Individuals with longer pre-study LVET (determined by a median split on pre-study LVET) tended to have poorer performance during TSD and stress. Resilient and vulnerable groups (determined by a median split on average TSD performance) showed significantly different profiles of SV, HR, CI, and LVET. Importantly, LVET at pre-study, but not other hemodynamic measures, reliably differentiated neurobehavioral performance during TSD and stress, and therefore may be a biomarker. Future studies should investigate whether the non-invasive marker, LVET, determines risk for adverse health outcomes.

## Introduction

Chronic sleep deprivation is a significant public health issue and is associated with multiple adverse health risks such as cardiovascular disease, obesity, diabetes, cancer, and overall morbidity and mortality (Ferrie et al., 2007; Gallicchio and Kalesan, 2009; Mullington et al., 2009). For many individuals, sleep loss increases self-rated sleepiness and deficits in sustained attention (Banks and Dinges, 2007; Goel et al., 2009; Brieva et al., 2021; Casale et al., 2021; Yamazaki et al., 2021a,b). However, large, highly replicable, phenotypic individual differences occur in response to sleep deprivation, whereby some individuals are vulnerable, and others are resilient to sleep loss (Van Dongen et al., 2004; Goel, 2017; Yamazaki and Goel, 2020; Brieva et al., 2021; Casale et al., 2021; Yamazaki et al., 2021a,b). These inter-individual differences are robust for common types of sleep loss, such as total sleep deprivation (TSD) and chronic sleep restriction (SR) (Dennis et al., 2017; Yamazaki and Goel, 2020), persisting across months and years (Dennis et al., 2017), but do not differ between various subgroups, such as age, sex, race, and body mass index (Yamazaki and Goel, 2020).

The sleep and circadian systems are tightly integrated with—and can markedly affect—the cardiovascular (CV) system. CV variables such as stroke volume (SV), heart rate (HR), cardiac output, blood pressure (BP), left ventricular ejection time (LVET), and vascular resistance all display diurnal variations (Cugini et al., 1993; Morris et al., 2013; Thosar et al., 2018). During non-rapid eye movement (non-REM) sleep, HR and BP decrease from greater parasympathetic activity, while during REM sleep, there is a shift toward greater sympathetic activity; moreover, sleep loss results in less parasympathetic and greater sympathetic activity (Kato et al., 2000; Henelius et al., 2014; Tobaldini et al., 2017). During TSD and SR, CV measures such as SV (Lü et al., 2018), HR (Kato et al., 2000; Meier-Ewert et al., 2004; Zhong et al., 2005; Sauvet et al., 2010; Sunbul et al., 2014; Lü et al., 2018; Bourdillon et al., 2021), cardiac index (CI) (Sunbul et al., 2014), BP (Kato et al., 2000; Muenter et al., 2000; Meier-Ewert et al., 2004; Zhong et al., 2005; Mullington et al., 2009; Sauvet et al., 2010; Lü et al., 2018; Bourdillon et al., 2021; Bozer et al., 2021; Cernych et al., 2021), and vascular resistance (Kato et al., 2000; Lü et al., 2018) have shown inconsistent changes, with some studies reporting alterations, while others show no changes. To our knowledge, no prior study has examined changes in LVET during sleep deprivation. LVET is strongly positively correlated with SV in healthy participants and is altered in patients with cardiovascular disorders (CVD), such as heart failure (Reant et al., 2010). Heart failure patients show disrupted sleep and are at high risk for obstructive sleep apnea syndromes (Pak et al., 2019).

Similarly, both acute and prolonged stress alter CV measures and increase risk for CVD, such as hypertension and coronary artery calcification (Turner et al., 2020). The Trier Social Stress Test (TSST) (Kirschbaum et al., 1993) is a well-validated acute psychological stressor that decreases SV and LVET and increases HR, cardiac output, BP, and vascular resistance (Allen et al., 2014; Jayasinghe et al., 2017). Notably, the combination of sleep loss and various stress conditions also increases BP (Kato et al., 2000; Bozer et al., 2021). To our knowledge, the time course of changes in SV, HR, CI, LVET, BP, and systemic vascular resistance index (SVRI) across sleep loss and psychological stress has not thus far been investigated.

Given the synergistic effects of sleep loss and stress on the CV system, CV measures are potential novel candidate biomarkers that have not yet been investigated to identify individuals who are resilient or vulnerable to these potent stressors. We determined whether the combination of sleep loss and psychological stress affects a comprehensive set of CV measures and whether these measures differentiate vulnerable and resilient individuals before and in response to TSD and stress, which is particularly important to consider in applied settings, where both are commonly experienced (Barger et al., 2014; Cromwell et al., 2021; Mhatre et al., 2021). We hypothesized the following: (1) TSD and stress would impair neurobehavioral performance; (2) CV measures at pre-study would identify individual differences in response to TSD and stress; (3) TSD and stress would alter CV measures; and (4) resilient and vulnerable individuals would show differential patterns of change in CV measures.

## Materials and Methods

### Participants

The Human Research Program Human Exploration Research Analog (HERA) is a high-fidelity space analog isolation facility located in Johnson Space Center in Houston, TX, United States. We studied 32 healthy adults (ages 27–53; mean age ± standard deviation [*SD*], 35.1 ± 7.1 years, 14 females) in this highly controlled facility. Groups of four participants at a time partook in one of the four HERA 14-day studies or one of the four 30-day studies. Participants were thoroughly screened by the National Aeronautics and Space Administration (NASA) and were required to pass a drug screen and a physical exam ensuring they were in excellent health with no history of CV, neurological, gastrointestinal, or musculoskeletal problems. The study was approved by the Institutional Review Boards of the NASA and of the University of Pennsylvania, and all protocol methods were carried out in accordance with approved guidelines and regulations. Participants provided written informed consent prior to inclusion in the study, which was in accordance with the Declaration of Helsinki. Participants received compensation for their participation in the protocol.

### Procedures

**FIGURE 1:**
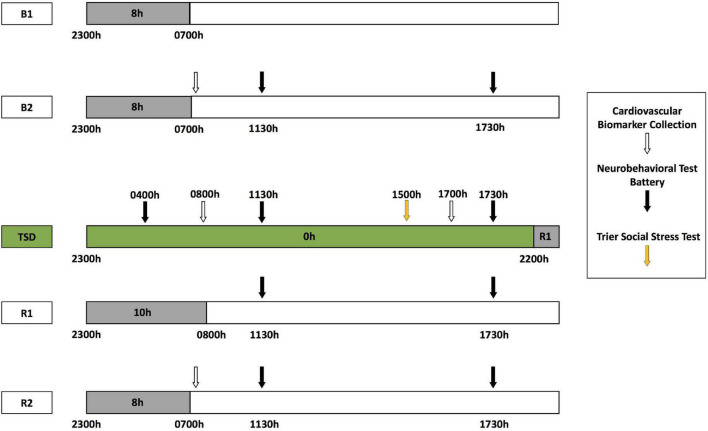
Five-day experimental protocol. Participants received two nights of baseline with 8-h time in bed (TIB) sleep opportunity (B1, B2; 2300—0700 h). Baseline cardiovascular (CV) measure collection (white arrows) occurred at 0700 h after the B2 night, followed by neurobehavioral test battery (NTB) administration at 1130 and 1730 h (black arrows). Following B2 daytime, participants experienced continued wakefulness for 39 h of total sleep deprivation (TSD, green block). NTB administration occurred at 0400 h during TSD, with CV measure collection at 0800 h and NTB administration at 1130 h. The modified Trier Social Stress Test (TSST, yellow arrow) was administered starting at 1500 h during the TSD day, with CV measure collection and NTB administration after completion at 1700 and 1730 h, respectively. Recovery sleep opportunities were 10 and 8 h TIB (R1 and R2, respectively). The NTB was administered at 1130 and 1730 h during R1 and R2, and CV measure collection occurred at 0700 h of R2.

During each HERA study, participants engaged in pre-study data collection, a 5-day experiment designed to induce stress and sleep deprivation and to measure neurobehavioral performance (Figure 1), and post-study data collection. The 5-day experiment consisted of 2 baseline nights [B1 and B2; 8-h time-in-bed (TIB), 2300—0700 h], followed by 39-h acute TSD during which participants remained awake. A modified TSST was conducted between 1500—1730 h on the day after the TSD night to induce psychosocial stress (described below). TSD was followed by a 10-h TIB recovery night (R1; 2200—0800 h), and a second 8-h TIB recovery night (R2; 2300—0700 h). Although fitness levels were not explicitly measured, all participants endured similar amounts of activity during the study, were confined to engaging in prescribed activities at specific times, and napping was prohibited during the experiment. Wrist actigraphy (Philips Respironics Healthcare, Bend, OR, United States) was used to measure total sleep time, sleep onset latency, and wake after sleep onset (Table 1). Actigraphic sleep data were analyzed as in our prior studies (Dennis et al., 2017; Moreno-Villanueva et al., 2018; Yamazaki and Goel, 2020; Brieva et al., 2021; Casale et al., 2021; Yamazaki et al., 2021a,b).

**TABLE 1:** Participant characteristics and actigraphic sleep data during the 5-day experiment (Mean ± SD).

		All participants	10-min PVT^a^ resilient	10-min PVT vulnerable	KSS^b^ resilient	KSS vulnerable
*N*		32	16	16	16	16
Sex (female/male)		14/18	7/9	7/9	6/10	8/8
Age		35.1 ± 7.15	35.4 ± 7.32	34.8 ± 7.19	35.1 ± 7.39	35.2 ± 7.14
Body surface area (m^2^)		1.84 ± 0.24	1.87 ± 0.27	1.83 ± 0.21	1.86 ± 0.29	1.84 ± 0.18
Baseline 1^c^	TST (min)^d^	405.8 ± 32.4	401.9 ± 35.7	409.4 ± 29.7	395.7 ± 35.4	416.5 ± 25.8
	SOL (min)^e^	11.6 ± 17.9	14.9 ± 24.8	8.50 ± 6.78	17.8 ± 23.0	5.00 ± 5.14
	WASO (min)^f^	37.1 ± 20.3	33.9 ± 21.8	40.1 ± 18.9	34.9 ± 16.5	39.5 ± 24.0
Baseline 2	TST (min)	402.6 ± 35.3	398.2 ± 38.4	407.0 ± 32.5	398.8 ± 39.4	406.4 ± 31.5
	SOL (min)	11.5 ± 24.9	16.6 ± 34.1	6.44 ± 7.68	12.3 ± 34.4	10.8 ± 9.49
	WASO (min)	38.2 ± 19.7	34.8 ± 14.3	41.7 ± 24.0	32.9 ± 14.4	43.5 ± 23.2
Total sleep deprivation	TST (min)	–	–	–	–	–
	SOL (min)	–	–	–	–	–
	WASO (min)	–	–	–	–	–
Recovery 1^c^	TST (min)	528.3 ± 69.4	517.7 ± 90.7	538.3 ± 41.8	534.9 ± 40.5	521.2 ± 92.0
	SOL (min)	1.81 ± 3.36	2.47 ± 4.27	1.19 ± 2.17	1.81 ± 3.92	1.80 ± 2.78
	WASO (min)	51.3 ± 47.3	44.1 ± 40.2	58.1 ± 53.5	59.6 ± 54.7	42.4 ± 37.7
Recovery 2^c^	TST (min)	390.3 ± 50.1	385.2 ± 45.0	395.0 ± 55.5	381.9 ± 55.7	399.1 ± 43.5
	SOL (min)	12.3 ± 13.4	12.7 ± 16.6	11.9 ± 10.0	13.7 ± 16.4	10.9 ± 9.51
	WASO (min)	47.3 ± 36.9	39.1 ± 20.0	55.0 ± 47.0	49.6 ± 47.2	44.9 ± 22.5

*^a^PVT, Psychomotor Vigilance Test; ^b^KSS, Karolinska Sleepiness Scale; ^c^N = 15 in the 10-min PVT resilient group and the KSS vulnerable group; ^d^TST, total sleep time; ^e^SOL, sleep onset latency; ^f^WASO, wake after sleep onset. Resilient and vulnerable groupings were based on a median split on average PVT performance or on average KSS scores during total sleep deprivation and psychological stress.*

### Cardiovascular Measure Collections

All echocardiogram and BP measures were collected under highly controlled conditions at six time points: pre-study, B2, the morning of TSD (TSD AM), the evening of TSD (TSD PM), R2, and post-study (Figure 1). All collections were completed at the same time each day (0800 h before eating), except for the TSD PM assessment, which was collected at 1730 h. Pre- and post-study collections occurred 1 day before and 4 or 5 days after the study, respectively, in the same location as collections during the 5-day experiment. All participants fasted for 10 h and for 5 h prior to all five AM collections and the one PM collection, respectively, to maintain consistency across the study and among participants.

#### Echocardiogram Procedures

One participant collected all cardiac ultrasound images on the other three participants during each study, and a second participant performed the collection on the primary collector. All collectors were highly trained by an echocardiogram specialist prior to the study and followed identical procedures at every time point.

Stroke volume, CI, LVET, and the peak-to-peak interval were derived from Doppler obtained via cardiac ultrasound imaging [GE Vivid q ultrasound system (General Electric Medical Systems, Milwaukee, WI, United States)] in a seated position at all time points (Haites et al., 1985; McLennan et al., 1986; Arbeille and Herault, 1998). Two-dimensional images of the left ventricular outflow tract (LVOT) were collected from each participant using a 5S-RS transducer. The LVOT was imaged from the parasternal long-axis view while the participants were semi-supine in a left lateral decubitus posture. Three to four, two-second cine-loops of dynamic motion of the LVOT were digitally saved. SV was collected utilizing a continuous wave (CW) pencil (Pedof) probe for Doppler interrogation. CW Doppler signals were taken from the ascending aorta at the suprasternal notch in a seated posture. Three 5-s cine-loop sweeps of CW Doppler data were collected and digitally stored as proprietary raw data.

Analysis of the digital data was conducted using Echo PAC PC (BT12) software (General Electric Medical Systems, Milwaukee, WI, United States). LVOT diameters were measured just proximal to the aortic valve leaflet insertion from three consecutive cine-loops at the maximum opening of the aortic valve. Five consecutive CW Doppler waveform profiles were traced to calculate the velocity time integral (VTI). The interval between each maximum peak on the Doppler spectral from the ascending aorta was used to calculate the peak-to-peak time in milliseconds (ms). This peak-to-peak time was used as a surrogate to the R–R interval to calculate HR. The duration of each beat was measured to determine LVET for each SV. The VTI and LVET were then transferred from the Echo PAC software to Excel to calculate SV, HR, and CI using the following formulas:

SV = (LVOT cross sectional area)*VTI
  HR = 60/(R−R interval)
  CI = [(SV*HR)/1,000]/body surface area

#### Blood Pressure and Systemic Vascular Resistance

Brachial systolic BP (SBP) and diastolic BP (DBP) were recorded using an Omron BP791IT 10 series Plus Automatic Blood Pressure Monitor with ComFit™ Cuff (Lake Forest, IL, United States) in a seated position on the non-dominant arm. Participants were seated for 3 min before BP collection. The average value of three consecutive readings, taken 1 min apart, was used for analyses. SVRI was calculated by assuming that central venous pressure was zero and by using the following equations (Klabunde, 2012; Norsk et al., 2015):

Mean arterial pressure = (SBP + 2*DBP)/3
  SVRI = mean arterial pressure/CI

### Neurobehavioral Performance

Each participant completed 11 precise computer-based neurobehavioral testing sessions during the study [Dell Latitude E5420 Laptops; Software: Windows XP; NTB custom reaction time (RT) testing software (Pulsar Informatics, Inc., Philadelphia, PA, United States)]. The neurobehavioral testing battery (NTB) was administered at 1130 and 1730 h each day of the 5-day experiment, with an additional test at 0400 h after a night of TSD (Figure 1). NTB sessions were administered during B1 for practice and were excluded from analyses. The NTB included the 10-min Psychomotor Vigilance Test (PVT) (Basner and Dinges, 2011), an objective behavioral attention test measuring the total number of lapses (RT > 500 ms) and errors (RT < 100 ms) and the Karolinska Sleepiness Scale (KSS) (Åkerstedt and Gillberg, 1990), measuring self-rated sleepiness. The PVT and KSS are highly sensitive and stable, well-validated measures to sleep loss (Basner and Dinges, 2011; Åkerstedt et al., 2014), that show robust individual differences without practice effects (Dennis et al., 2017; Yamazaki and Goel, 2020; Casale et al., 2021; Yamazaki et al., 2021a,b), and have been examined previously in a CV study (Henelius et al., 2014). Resilient and vulnerable individuals were determined by a median split on average PVT total lapses and errors and on average KSS scores from the three NTB sessions during TSD (Patanaik et al., 2015; Moreno-Villanueva et al., 2018; Caldwell et al., 2020). We dichotomized participants as such, since for initial examination and categorization of novel biomarkers, it is more suitable and applicable to create resilient and vulnerable groups (Chuah et al., 2009; Rocklage et al., 2009; Chee and Tan, 2010; Diekelmann et al., 2010; Patanaik et al., 2015; Yeo et al., 2015; Xu et al., 2016; Moreno-Villanueva et al., 2018; Caldwell et al., 2020; Salfi et al., 2020; Brieva et al., 2021; Casale et al., 2021; Yamazaki et al., 2021b), especially given our sample size. Systematic examination of various approaches and thresholds for assessing differential neurobehavioral vulnerability to sleep loss has also shown that median splits on averaged performance scores, rather than change from baseline or variance in scores, are consistent indicators of resilience and vulnerability during both sleep-deprived and well-rested periods (Brieva et al., 2021; Casale et al., 2021; Yamazaki et al., 2021b), thus further justifying our methods. Table 2 depicts each participant’s resilient or vulnerable status for PVT performance and for KSS scores.

**TABLE 2:** Resilient and vulnerable group categorizations for each participant (*N* = 32) for 10-min Psychomotor Vigilance Test (PVT) lapses and errors and Karolinska Sleepiness Scale (KSS) scores.

Participant ID	10-min PVT	KSS
001	V^a^	V
002	R^b^	V
003	R	R
004	V	R
005	V	V
006	R	V
007	R	R
008	V	R
009	R	R
010	V	V
011	V	V
012	V	R
013	V	V
014	R	R
015	V	V
016	V	V
017	R	R
018	V	V
019	R	V
020	R	V
021	R	V
022	V	V
023	V	V
024	V	R
025	R	R
026	R	R
027	R	R
028	R	R
029	R	R
030	V	V
031	R	R
032	V	R

*^a^V, vulnerable; ^b^R, resilient.*

*Resilient and vulnerable groupings were based on a median split on average PVT performance or on average KSS scores during total sleep deprivation and psychological stress.*

### Trier Social Stress Test

The TSST is a commonly used and validated test to experimentally induce psychosocial stress (Kirschbaum et al., 1993; Allen et al., 2014). It has been successfully modified and validated using a virtual, rather than a physical audience (Kelly et al., 2007; Ruiz et al., 2010; Helminen et al., 2021). A modified 30-min TSST, which consisted of several challenging interview questions regarding responses to TSD, including those related to performance, aptitude, motivation, and interactions with others, and several difficult cognitive tests, including a 3-min Stroop task and a 5-min calculation task involving counting backward aloud in 13-step sequences, was conducted with participants remotely via audio and a one-way video camera (Moreno-Villanueva et al., 2018).

### Statistical Analyses

All statistical analyses were performed using SPSS v22 (SPSS Inc., Chicago, IL, United States), with *p* < 0.05 considered statistically significant and all statistical tests were two-tailed. Descriptive statistics characterizing the sample and outcome measures, including mean, SD, and SEM, are indicated in the results, table, and figures. Prior studies have found normal distributions for the performance and CV measures examined in this study (Orme et al., 1999; Dennis et al., 2017; Moreno-Villanueva et al., 2018; Yamazaki and Goel, 2020), and other studies have employed appropriate statistics tests, such as ANOVAs and *t*-tests, accordingly (Kato et al., 2000; Muenter et al., 2000; Sunbul et al., 2014; Lü et al., 2018). Greenhouse–Geisser corrections for degrees of freedom were applied for all repeated measures (RM) analysis of variance (ANOVAs) for all analyses.

A median split on each hemodynamic measure during pre-study defined low and high or short and long (for LVET) pre-study groups. One-way ANOVAs or chi-square tests determined differences in pre-study low/high and short/long groups in age, sex, and body surface area (BSA). RMANOVAs evaluating PVT performance and KSS scores included the factors ‘group’ (low/high or short/long pre-study group) and ‘condition’ [baseline (averaged from the two B2 NTBs), TSD (averaged from the three TSD NTBs), and recovery (averaged from the four R1 and R2 NTBs)], and the interaction ‘condition*pre-study group.’ *Post hoc* comparisons with Bonferroni corrections were used to detect performance differences between conditions if a significant condition effect was detected (e.g., PVT performance at baseline vs. PVT performance at TSD in the entire sample). Bonferroni-corrected *p*-values are reported. *Post hoc* one-way ANOVAs assessed performance differences between pre-study groups at each condition when a significant interaction or group effect was detected (e.g., PVT performance at baseline in the short pre-study LVET group vs. PVT performance at baseline in the long pre-study LVET group). Spearman’s relative rank correlation assessed the relationship between average PVT and KSS responses during TSD.

A median split on average PVT performance and on average KSS scores during TSD and stress determined resilient and vulnerable groups (Patanaik et al., 2015; Moreno-Villanueva et al., 2018; Caldwell et al., 2020). One-way ANOVAs or chi-square tests determined differences between PVT and KSS resilient/vulnerable groups in age, sex, BSA, and actigraphic sleep characteristics across the study. RMANOVAs evaluating CV measures included the factors ‘group’ (resilient or vulnerable for PVT total lapses and errors or KSS scores) and ‘condition’ (pre-study, baseline, TSD AM, TSD PM, recovery, and post-study), and the interaction ‘condition*PVT/KSS group.’ *Post hoc* analyses with Bonferroni corrections compared each condition when there was a significant condition effect (e.g., HR at baseline vs. HR at TSD AM in the entire sample). Bonferroni-corrected *p*-values are reported. *Post hoc* one-way ANOVAs assessed CV differences between groups at each condition when there was a significant interaction or significant group effect (e.g., HR at baseline in the PVT resilient group vs. HR at baseline in the PVT vulnerable group). One participant was withdrawn from the study during R1 but returned for post-study data collection. All RMANOVAs did not include this individual’s data (*N* = 31) and all recovery *post hoc* comparisons did not include this individual’s data (*N* = 31); however, otherwise this individual’s data were included in analyses to maximize statistical power (*N* = 32).

## Results

### Participant Characteristics

There were no significant differences between resilient and vulnerable groups defined by PVT total lapses and errors or KSS scores in age [*F*(1) = 0.00–0.06, *p* = 0.809–0.962], sex [χ^2^(1) = 0.00–0.51, *p* = 0.476–1.000], or BSA [*F*(1) = 0.05–0.27, *p* = 0.608–0.826]. These groups also did not significantly differ in actigraphic total sleep time, sleep onset latency, or wake after sleep onset during the 5-day experiment [*F*(1) = 0.00–3.47, *p* = 0.073–0.992], except that the KSS vulnerable group had a small, but significantly shorter sleep onset latency at B1 than the KSS resilient group, [*F*(1) = 4.38, *p* = 0.045, difference between KSS resilient and KSS vulnerable groups = 12.75 min] but not at B2 [*F*(1) = 0.026, *p* = 0.873], which was the night before TSD. Table 1 shows actigraphic data for the entire sample, for 10-min PVT resilient and vulnerable groups, and for KSS resilient and vulnerable groups—overall, these data indicate the participants were healthy sleepers. In addition, pre-study low and high or short and long (for LVET) defined groups did not significantly differ in age [*F*(1) = 0.00–1.08, *p* = 0.307–1.000], sex [χ^2^(1) = 0.00–2.03, *p* = 0.154–1.000], or BSA [*F*(1) = 0.18–2.94, *p* = 0.097–0.676], except that the pre-study SBP, DBP, and SVRI-defined groups differed by sex composition, with more females in the low pre-study SBP (*N* = 12) and DBP (*N* = 11) groups and more females (*N* = 10) in the low pre-study SVRI group [χ^2^(1) = 4.57–12.70, *p* = 0.000–0.033]. In addition, the pre-study SVRI groups differed by BSA composition, with a greater BSA in the high pre-study SVRI group [*F*(1) = 7.895, *p* = 0.009].

### Sleep Loss and Stress Induced Neurobehavioral Deficits

**FIGURE 2:**
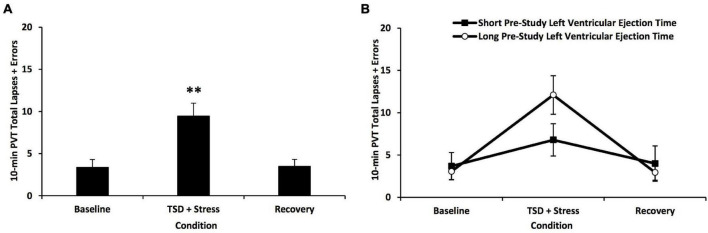
Changes in 10-min Psychomotor Vigilance Test (PVT) total lapses and errors in all participants and defined by a median split on pre-study left ventricular ejection time (LVET) to total sleep deprivation (TSD) and psychological stress. **(A)** TSD and psychological stress produced a significant increase in 10-min PVT lapses and errors; there were large individual differences in neurobehavioral responses, whereby some individuals were classified as resilient, and others were classified as vulnerable. PVT total lapses and errors returned to baseline levels with recovery. **(B)** Comparison of PVT total lapses and errors across the 5-day experiment in short vs. long pre-study LVET (defined by a median split on pre-study LVET values, using RMANOVA). There was a significant condition*pre-study group interaction. *Post hoc* analyses revealed a trend toward a difference in PVT performance between the short pre-study and the long pre-study LVET groups during the TSD + stress condition, whereby the short pre-study LVET group had better PVT performance than the long pre-study LVET group [*F*(1) = 3.185, *p* = 0.087]. For **(A,B)**, the baseline point is an average of two neurobehavioral test batteries (NTBs) during B2, the TSD + stress point is an average of three NTBs during TSD, and the recovery point is an average of four NTBs during recovery days 1 and 2 (pre-study is not depicted because the NTB was not administered during pre-study). For **(A)**, *N* = 31 for the recovery point due to one participant withdrawn during recovery; all other points are *N* = 32. For **(B)**, *N* = 15 in the recovery point of the long pre-study LVET group due to the same participant withdrawn during recovery; all other data points are *N* = 16. ^**^p < 0.001. Data are mean ± SEM.

**FIGURE 3:**
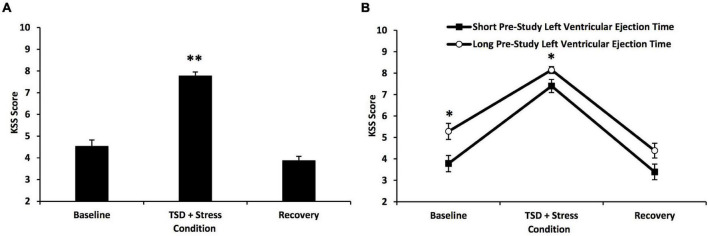
Changes in Karolinska Sleepiness Scale (KSS) scores in all participants and defined by a median split on pre-study left ventricular ejection time (LVET) to total sleep deprivation (TSD) and psychological stress. **(A)** TSD and psychological stress produced a significant increase in KSS scores; there were large individual differences in scores, whereby some individuals were classified as resilient, and others were classified as vulnerable. KSS scores returned to baseline levels with recovery. **(B)** Comparison of KSS score changes across the 5-day experiment in short pre-study vs. long pre-study LVET groups (defined by a median split on pre-study LVET values, using RMANOVA). There was a significant overall between-subject effect, whereby the long pre-study LVET group reported, on average, significantly greater sleepiness than the short pre-study LVET group. *Post hoc* analyses revealed that the long pre-study LVET group reported significantly greater sleepiness at baseline and during TSD + stress. For **(A,B)**, the baseline point is an average of two neurobehavioral test batteries (NTBs) during baseline day 2, the TSD + stress point is an average of three NTBs during TSD, and the recovery point is an average of four NTBs during recovery days 1 and 2 (pre-study is not depicted because the NTB was not administered during pre-study). For **(A)**, *N* = 31 for the recovery point due to one participant withdrawn during recovery; all other data points are *N* = 32. For **(B)**, *N* = 15 in the recovery point of the long pre-study LVET group due to the same participant withdrawn during recovery; all other data points are *N* = 16. *p < 0.05, ^**^p < 0.001. Data are mean ± SEM.

Total sleep deprivation and psychological stress significantly affected 10-min PVT performance (Figure 2A) and KSS scores (Figure 3A) [*F*(1.22–1.89, 35.42–54.91) = 28.49–185.43, *p*’s < 0.001]. As expected, there were large individual differences in neurobehavioral responses to TSD and stress: average 10-min PVT lapses and errors ranged from 1.33 to 36.3 (resilient group mean ± SD: 4.1 ± 1.82; vulnerable group mean ± SD: 14.79 ± 9.62; Figure 2A); average KSS scores ranged from 5.00 to 9.00 (resilient group mean ± SD: 7.02 ± 0.91; vulnerable group mean ± SD: 8.52 ± 0.40; Figure 3A). PVT performance and KSS scores during TSD did not significantly correlate with each other (ρ = 0.29, *p* = 0.625). Both PVT performance and KSS scores returned to baseline levels with recovery (Figures 2A, 3A).

### Psychomotor Vigilance Test and Karolinska Sleepiness Scale Profiles Across Total Sleep Deprivation and Stress in Low vs. High or Short vs. Long Pre-study Cardiovascular Groups

#### Left Ventricular Ejection Time

The short vs. long pre-study LVET groups showed a significant condition*pre-study LVET group interaction across PVT performance during the 5-day experiment [*F*(1.31, 38.10) = 11.59, *p* = 0.001] (Figure 2B). *Post hoc* analyses revealed a trend toward a difference in PVT performance between the short pre-study and the long pre-study LVET groups during the TSD and stress condition, whereby the short pre-study LVET group had better PVT performance than the long pre-study LVET group [*F*(1) = 3.185, *p* = 0.087]. There were no significant differences between the short and long pre-study LVET group at baseline or recovery. The short vs. long pre-study LVET groups did not show a significant condition*pre-study group interaction across KSS scores during the 5-day experiment [*F*(1.89, 54.91) = 1.58, *p* = 0.216] (Figure 3B). There was a significant overall group difference in KSS scores for the short vs. long pre-study LVET analysis [*F*(1) = 6.65, *p* = 0.015]: the long pre-study LVET group reported, on average, greater sleepiness than the short pre-study LVET group. *Post hoc* analyses revealed that the short and long pre-study LVET groups differed significantly on their KSS scores at baseline and TSD + stress (Figure 3B): the long pre-study group reported greater sleepiness than the short pre-study group at both conditions [*F*(1) = 4.78–7.89, *p* = 0.009–0.037].

#### Seated Stroke Volume, Heart Rate, Cardiac Index, Blood Pressure, and Systemic Vascular Resistance Index

For all other CV measures, the low vs. high pre-study groups did not show any significant condition*PVT group or condition*KSS group interactions [*F*(1.22–1.89, 35.42–54.91) = 0.17–3.02, *p* = 0.084–0.834] or overall PVT or KSS group differences [*F*(1) = 0.01–2.51, *p* = 0.124–0.920].

### Cardiovascular Measure Profiles Across Total Sleep Deprivation and Stress Resilient vs. Vulnerable Groups

All CV measures were within normal, healthy adult ranges (Klabunde, 2012; Cattermole et al., 2017; Shaffer and Ginsberg, 2017).

#### Seated Stroke Volume

**FIGURE 4:**
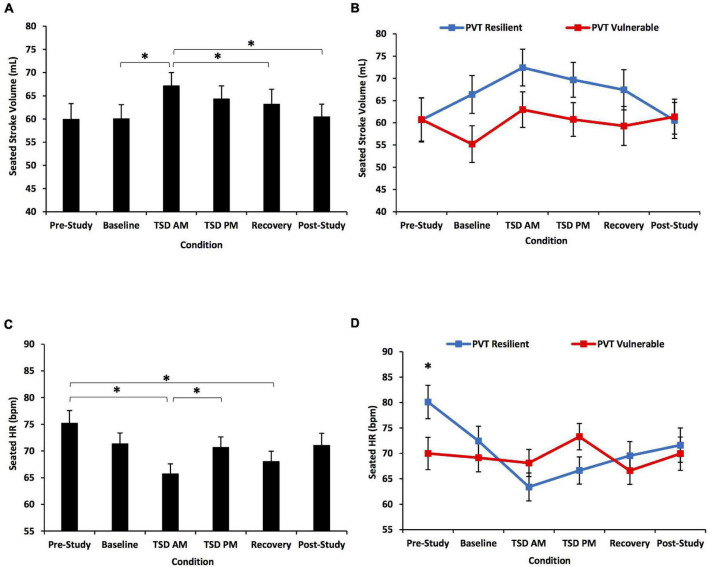
Seated stroke volume (SV) and heart rate (HR) changes across the study for all participants and defined by a median split on 10-min Psychomotor Vigilance Test (PVT) total lapses and errors. The top row shows SV changes across the study: **(A)** for all participants and **(B)** divided by a resilient or vulnerable median split on PVT total lapses and errors. There was a significant condition effect and condition*PVT group interaction. The bottom row shows HR changes across the study: **(C)** for all participants and **(D)** divided by a resilient or vulnerable median split on PVT total lapses and errors. There was a significant condition effect and condition*PVT group interaction, and the PVT resilient group had significantly greater HR than the vulnerable group at pre-study. *N* = 31 for recovery points, *N* = 32 for all other points in **(A,C)**; *N* = 15 in the resilient recovery point in **(B,D)** due to one withdrawn participant during recovery; *N* = 16 for all other data points. *p < 0.05. Data are mean ± SEM.

**FIGURE 5:**
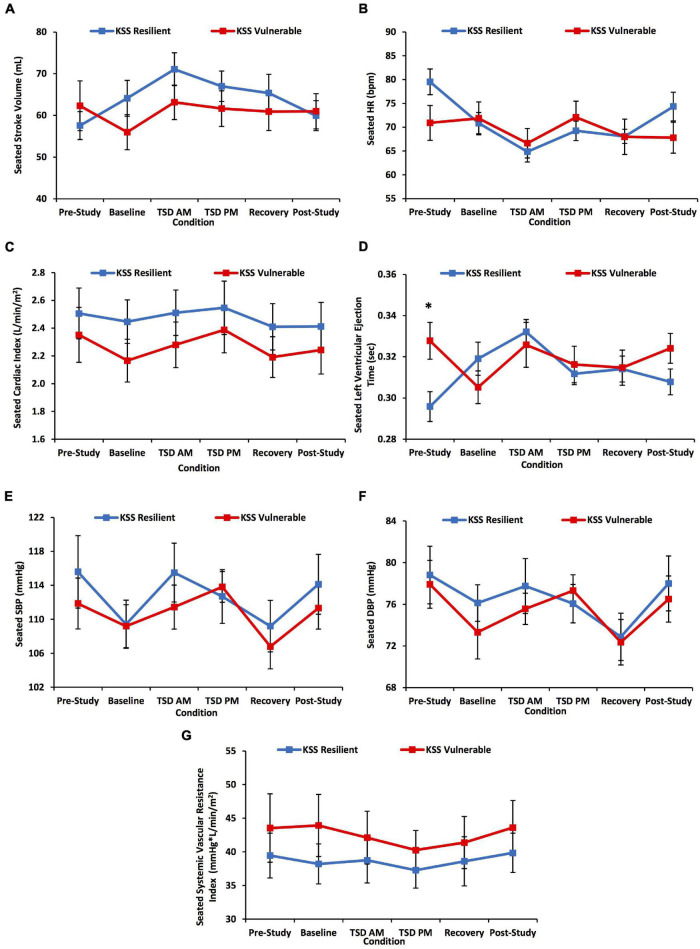
Seated cardiovascular biomarker changes across the study defined by a median split on Karolinska Sleepiness Scale (KSS) scores. The change across the study was divided by a resilient or vulnerable median split on KSS scores for **(A)** stroke volume (SV); **(B)** heart rate (HR); **(C)** cardiac index (CI); **(D)** left ventricular ejection time (LVET); **(E)** systolic blood pressure (SBP); **(F)** diastolic blood pressure (DBP); and **(G)** systemic vascular resistance index (SVRI). There was a significant time*KSS group interaction for SV, HR, and LVET. Pre-study LVET was significantly longer in KSS vulnerable than resilient participants. *N* = 15 in the vulnerable recovery point in **(A–G)** due to one withdrawn participant during recovery. All other data points are *N* = 16. *p < 0.05. Data are mean ± SEM.

SV showed a significant condition effect [*F*(3.09, 89.70) = 3.37, *p* = 0.021]: TSD AM SV was significantly greater than baseline, recovery, and post-study SVs (*p* = 0.001–0.047) (Figure 4A). SV also showed a significant condition*PVT group interaction [*F*(3.09, 89.70) = 2.77, *p* = 0.045], but no significant differences between groups at any condition or an overall group effect [*F*(1) = 0.05–2.67, *p* = 0.113–0.817] (Figure 4B). SV also showed a significant condition*KSS group interaction [*F*(3.22, 93.50) = 3.18, *p* = 0.025] (Figure 5A).

#### Seated Heart Rate

There was a significant condition effect for seated HR across the study [*F*(4.03, 116.90) = 5.96, *p* < 0.001] (Figure 4C): pre-study HR was significantly higher than TSD AM and recovery HR, and TSD AM HR was significantly lower than TSD PM HR (*p* = 0.003–0.032). There was a significant condition*PVT group interaction [*F*(4.03, 116.90) = 5.65, *p* < 0.001], and PVT resilient individuals had significantly higher HR than PVT vulnerable individuals at pre-study [*F*(1) = 5.58, *p* = 0.025] (Figure 4D). There was no significant overall group effect [*F*(1) = 0.11, *p* = 0.747]. HR also showed a significant condition*KSS group interaction [*F*(3.72, 107.94) = 3.55, *p* = 0.011] (Figure 5B).

#### Seated Cardiac Index

**FIGURE 6:**
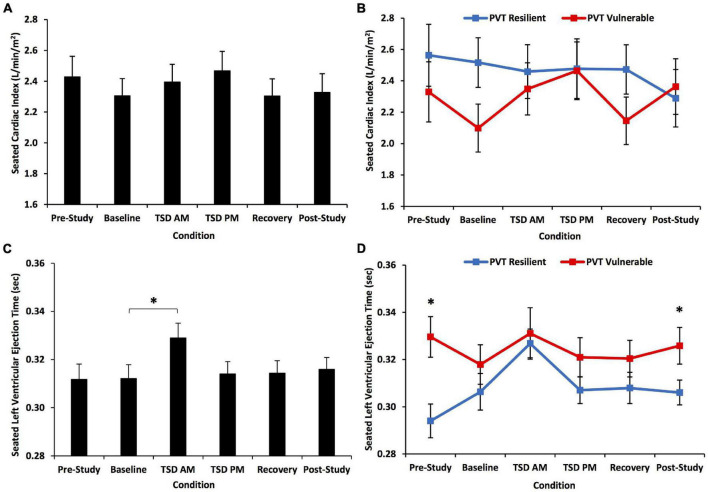
Seated cardiac index (CI) and left ventricular ejection time (LVET) changes across the study for all participants and defined by a median split on 10-min Psychomotor Vigilance Test (PVT) total lapses and errors. The top row shows CI changes across the study: **(A)** for all participants and **(B)** divided by a resilient or vulnerable median split on PVT total lapses and errors. There was a significant condition*PVT group interaction for CI. The bottom row shows LVET changes across the study: **(C)** for all participants and **(D)** divided by a resilient or vulnerable median split on PVT total lapses and errors. There was a significant condition effect for LVET. The condition*PVT group interaction was at significance, and the PVT vulnerable group had significantly longer LVET than the PVT resilient group at pre-study and post-study. *N* = 31 for recovery points, *N* = 32 for all other points in **(A,C)**; *N* = 15 in the resilient recovery point in **(B,D)** due to one withdrawn participant during recovery; *N* = 16 for all other data points. *p < 0.05. Data are mean ± SEM.

CI did not show a significant condition effect across the study [*F*(3.43, 99.38) = 1.70, *p* = 0.164] (Figure 6A). However, CI showed a significant condition*PVT group interaction [*F*(3.43, 99.38) = 2.88, *p* = 0.033], but no significant differences in CI between the resilient and vulnerable groups at any condition or an overall group effect [*F*(1) = 0.00–3.80, *p* = 0.061–0.982] (Figure 6B). CI also did not show a significant condition*KSS group effect [*F*(3.51, 101.69) = 0.27, *p* = 0.873] (Figure 5C).

#### Seated Left Ventricular Ejection Time

LVET showed a significant condition effect across the study [*F*(4.39, 127.36) = 2.74, *p* = 0.027] (Figure 6C): baseline LVET was significantly shorter than that at TSD AM (*p* = 0.022). The condition*PVT group interaction for LVET was at significance [*F*(4.39, 127.36) = 2.32, *p* = 0.055], and PVT vulnerable individuals had significantly longer LVET than resilient individuals at pre-study and post-study [*F*(1) = 4.50–10.18, *p* = 0.003–0.042] (Figure 6D). There was no significant overall group effect [*F*(1) = 3.41, *p* = 0.075]. LVET also showed a significant condition*KSS group interaction [*F*(4.30, 124.61) = 6.35, *p* < 0.001]: the KSS vulnerable group had significantly longer LVET than the resilient group at pre-study [*F*(1) = 7.68, *p* = 0.009] (Figure 5D).

#### Seated Blood Pressure

**FIGURE 7:**
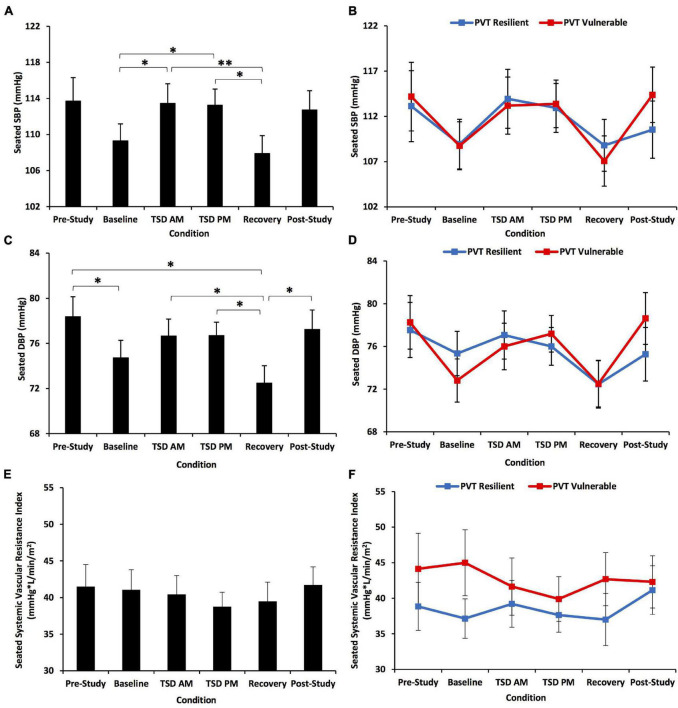
Seated systolic BP (SBP), diastolic BP (DBP), and systemic vascular resistance index (SVRI) changes across the study for all participants and defined by a median split on 10-min Psychomotor Vigilance Test (PVT) total lapses and errors. The first row shows SBP changes across the study: **(A)** for all participants and **(B)** divided by a resilient or vulnerable median split on PVT total lapses and errors. There was a significant condition effect for SBP. The middle row shows DBP changes across the study: **(C)** for all participants and **(D)** divided by a resilient or vulnerable median split on PVT total lapses and errors. There was a significant condition effect for DBP. The bottom row shows SVRI changes across the study: **(E)** for all participants and **(F)** divided by a resilient or vulnerable median split on PVT total lapses and errors. There was no significant condition effect across the study for SVRI. There were also no significant condition*PVT group interactions for SBP, DBP, or SVRI. *N* = 31 for recovery points, *N* = 32 for all other points in **(A,C,E)**; *N* = 15 in the resilient recovery point in **(B,D,F)** due to one withdrawn participant during recovery; *N* = 16 for all other data points. *p < 0.05, ^**^p < 0.001. Data are mean ± SEM.

SBP showed a significant condition effect across the study [*F*(3.41, 98.96) = 5.85, *p* = 0.001] (Figure 7A): baseline SBP was significantly lower than TSD AM and TSD PM SBP (*p* = 0.003–0.026), and TSD AM and TSD PM SBP were significantly higher than recovery SBP (*p* = 0.000–0.031). There was no significant condition*PVT group interaction [*F*(3.41, 98.96) = 0.85, *p* = 0.484] or significant overall group effect [*F*(1) = 0.01, *p* = 0.913] (Figure 7B). DBP showed a significant condition effect across the study [*F*(4.17, 120.95) = 5.23, *p* = 0.001] (Figure 7C): pre-study DBP was significantly higher than baseline and recovery DBP, and TSD AM, TSD PM, and post-study DBP were all significantly higher than recovery DBP (*p* = 0.005–0.034). There was no significant condition*PVT group interaction [*F*(4.17, 120.95) = 1.25, *p* = 0.283] or significant overall group effect [*F*(1) = 0.01, *p* = 0.911] (Figure 7D). There were no significant condition*KSS group interactions for SBP or DBP [*F*(3.35–4.27, 97.11–123.83) = 0.88–1.01, *p* = 0.406–0.467] (Figures 5E,F).

#### Seated Systemic Vascular Resistance Index

SVRI did not show a significant condition effect across the study [*F*(3.71, 107.68) = 1.01, *p* = 0.401] (Figure 7E). There was no significant condition*PVT group interaction [*F*(3.71, 107.68) = 1.67, *p* = 0.167], or significant overall group effect [*F*(1) = 0.76, *p* = 0.390] (Figure 7F). There also was no significant condition*KSS group interaction for SVRI [*F*(3.82, 110.76) = 0.25, *p* = 0.900] (Figure 5G).

## Discussion

Pre-study LVET differentiated individuals who were resilient or vulnerable during sleep loss and psychosocial stress, whereby individuals with shorter LVET at pre-study had better psychomotor vigilant attention performance during the combination of TSD and psychosocial stress than individuals with longer LVET at pre-study. Moreover, resilient individuals had significantly greater pre-study HR and shorter pre-study LVET than vulnerable individuals. Resilient individuals also had greater SV and CI from baseline through recovery than vulnerable individuals. We show for the first time that LVET differentiates neurobehavioral performance resiliency and vulnerability during TSD and psychological stress. LVET warrants further investigation as a possible biomarker.

For the first time, we show that individuals who had short and long pre-study LVET showed differential changes in these measures across the experiment, evinced by a significant time*pre-study group interaction (Figure 2A). These novel results are parallel to—and thus validate—our finding that the PVT resilient group had significantly shorter LVET at pre-study and post-study than the PVT vulnerable group. The PVT resilient group also had significantly greater HR at pre-study than the PVT vulnerable group. Since LVET is a valid identifier of performance resilience and vulnerability, it may be a key target for identifying and mitigating the numerous negative repercussions of decrements induced by sleep loss and stress (Van Dongen et al., 2006; Howard et al., 2014).

Cardiac index and SVRI did not significantly change with TSD and psychological stress, consistent with prior studies (Kato et al., 2000; Lü et al., 2018). However, seated SV, HR, LVET, and BP all significantly changed with sleep loss and psychological stress as has been shown in some studies (Kato et al., 2000; Meier-Ewert et al., 2004; Zhong et al., 2005; Sauvet et al., 2010; Sunbul et al., 2014; Lü et al., 2018; Bourdillon et al., 2021; Bozer et al., 2021; Cernych et al., 2021), but not others (Kato et al., 2000; Sauvet et al., 2010; Bourdillon et al., 2021; Bozer et al., 2021). Discrepancies in study findings may be due to differences in the severity of TSD and/or stress conditions or in hemodynamic collection methods such as the angle dependency of Doppler. However, R–R timing measures derived from Doppler are independent of angle correction and similar to direct R–R interval measures derived from electrocardiography or applanation tonometry.

Notably, standing SV, HR, CI, and LVET were also measured 3 min after the seated measures but were not reported in the main results. SV, LVET, and BP were all lower than their respective seated measures, an expected finding considering volume shifts in participants who had been fasting. Inversely, HR increased as a compensatory mechanism to adequate perfusion, thus decreasing R–R interval (Klabunde, 2012). Additionally, with a few minor exceptions, there were no significant differences in changes from seated to standing measures at any condition between the resilient and vulnerable groups, reflecting the fact that all participants were at equivalent baseline volume status. The differences between the vulnerable and resilient groups can thus be attributed to inherent group differences in response to TSD and stress, underscoring the validity of the data collected. Also, of note, the PVT and KSS performance results are similar to findings obtained in our sleep deprivation studies conducted in the laboratory (Dennis et al., 2017; Yamazaki and Goel, 2020; Casale et al., 2021; Yamazaki et al., 2021a,b).

We hypothesize that individuals with shorter pre-study LVET, who may be resilient to TSD and stress, may have a basal CV system that is in an “overdrive” aroused state marked by greater sympathetic activity. However, when these individuals experience stressful situations, such as acute TSD and psychological stress, parasympathetic activity increases, bringing autonomic balance into an optimal state and moderating the toll of these potent stressors, thereby maintaining performance. Additionally, it is possible that differences in preload and after load may be mediating the differences in CV status between the resilient and vulnerable groups. Future studies should test these hypotheses. Furthermore, past studies support our hypothesis regarding optimization of autonomic balance to maintain performance. Barber et al. (2020) found similar results to those in our study: participants with longer mean interbeat interval, thus lower HR, and greater parasympathetic activity had better performance during an attention task and cortical activity increased with elevated parasympathetic arousal during this task. Additionally, a study by Chua et al. (2014) found that individuals with lower HR during TSD had worse performance during this time, which is contrary to our findings and those of Barber et al. (2020). None of these studies implemented both TSD and stress, and it is possible that the synergistic combination of stressors changes autonomic balance and its effects on performance compared to a single stressor. Future research is needed to systematically explore the optimization of autonomic balance in order to maintain neurobehavioral performance.

Notably, the SV, HR, and LVET changes across the study conditions in PVT resilient and vulnerable groups were similar to those in KSS resilient and vulnerable groups, as evinced by significant time*PVT/KSS group interactions for each CV measure. This is despite the fact that the PVT, a behavioral attention measure, and the KSS, a self-reported sleepiness measure, represent two vastly different neurobehavioral performance domains (Dennis et al., 2017; Yamazaki and Goel, 2020). Moreover, those individuals categorized as resilient by the PVT were not necessarily categorized as resilient by KSS, as shown in this and other studies (Dennis et al., 2017; Yamazaki and Goel, 2020). Our findings therefore suggest a common underlying mechanism of resilience, which may be linked by the hemodynamic measures examined in this study. Further research should explore how CV measures may be related to or differentiate resilience and vulnerability using objective performance measures other than behavioral attention (e.g., memory, cognitive throughput, etc.), since, similar to the lack of association between objective and subjective measures (Dennis et al., 2017; Yamazaki and Goel, 2020), the vulnerability to such tasks during sleep loss is not necessarily related to PVT performance (Frey et al., 2004; Tkachenko and Dinges, 2018).

During TSD AM, which may be the most difficult time to remain alert due to the circadian nadir (Goel et al., 2009), a sharp increase in SV and decrease in HR from baseline occurred in resilient participants. These inverse changes maintained the stability of CI in the resilient group. However, in the vulnerable group, although SV increased from baseline by a similar magnitude as in resilient participants, it was not matched by a decrease in HR. Thus, CI in vulnerable participants fluctuated across the study, and perhaps put those individuals at risk for cognitive decrements. Similarly, the KSS resilient participants had a large increase in SV from pre-study to TSD AM and also maintained CI stability. It is unclear whether HR or SV drove the change in the other measure and maintained CI in the resilient group.

Of note, post-study HR failed to return to pre-study levels in the PVT resilient group. Yang et al. (2019) showed that at least three nights of recovery sleep are needed to fully restore autonomic functions that regulate the CV system. This may partially explain the lack of HR recovery in the PVT resilient group, though further research is needed to determine why this pattern was not observed in the vulnerable group. Additionally, our study’s semi-isolated environment may have affected recovery length by changing brain structures in resilient individuals (Cromwell et al., 2021; Mhatre et al., 2021). Stahn et al. (2019) found that individuals had decreased brain and gray matter volume at the end of a 14-month isolation period, which recovered to pre-study levels 1.5 months after the end of the isolation. These structural changes may have occurred in our study and induced changes to autonomic control of the CV system, albeit within a shorter time frame. Our larger sample size may have provided more power to detect different CV recovery lengths in resilient versus vulnerable individuals, that were dependent on structural changes from isolation. Future studies are needed to examine this possibility.

Similarly, further research is also needed regarding the underlying correlates and neural dynamics of differential vulnerability to sleep loss, particularly in relation to various neurobehavioral measures and biomarkers. Previous work has identified the brain regions that are recruited by certain neurobehavioral metrics and determined how sleep deprivation affects these associations. For example, the PVT has been found to primarily recruit regions involved in vigilant attention (i.e., the prefrontal cortex, the inferior parietal cortex, the motor cortex, and the visual cortex) (Nasrini et al., 2020; Smith et al., 2021) and the KSS has been shown to primarily recruit regions involved in attention and sensory transmission (i.e., the thalamus and the right middle frontal gyrus) (Sun et al., 2020; Motomura et al., 2021). Though these findings are promising, more research is needed to identify reliable neural signatures of neurobehavioral resilience and vulnerability to sleep deprivation (Chee and Tan, 2010; Li et al., 2020; Zhu et al., 2020).

There was a significant condition effect across the study for both SBP and DBP, with increases in BP during TSD AM and PM, confirming that sleep deprivation and psychosocial stress have a detrimental effect on BP. However, resilient and vulnerable individuals did not have differential patterns in BP change across the study. The lack of differences may be due to the resilient and vulnerable groups maintaining BP by different mechanisms. The resilient group may have increased BP by increasing SV and LVET efficiency, while the vulnerable group may have used mechanisms other than SVRI to maintain BP. Since BP is prone to moment-to-moment fluctuations, we may not have been able to detect the differences between resilient and vulnerable individuals across the study (O’Brien et al., 2003). Thus, in our study, BP was not a valid measure to identify and differentiate resilient versus vulnerable individuals to TSD and stress.

Notably, all the CV measures we collected in this study showed a time-of-day effect (Cugini et al., 1993; Morris et al., 2013; Thosar et al., 2018). The changes in most of these measures from TSD AM to TSD PM in both the resilient and vulnerable groups were generally of the same magnitude. Thus, the resilient and vulnerable groups were likely not differentially affected by sleep loss and stress in terms of time-of-day, attributing the varying changes in resilient and vulnerable groups to other mechanisms.

Resilient and vulnerable groups showed differential cardiac reactivity to the stress of sleep deprivation and psychological stress conditions. Acute psychological stress typically increases HR and BP (Allen et al., 2014), yet there are individual differences in cardiac stress reactivity and differential consequences. Blunted reactivity to psychological stress is associated with obesity, self-reported negative health, and depression (Carroll et al., 2017; Turner et al., 2020). Exaggerated reactivity also has adverse long-term consequences to health via development of CVD (Carroll et al., 2017; Turner et al., 2020); however, in the case of performance during sleep loss and stress, exaggerated cardiac reactivity seems to be beneficial. This is exemplified by the resilient group’s more drastic increases in SV and LVET and decrease in HR from pre-study to both TSD AM and TSD PM than in the vulnerable group. The resilient group’s exaggerated response to sleep deprivation alone and in combination with the TSST may protect against attentional impairment. Thus, there may be a trade-off to resilience and vulnerability: vulnerable individuals may not perform well when sleep deprived but may have less risk for future adverse health outcomes because of their less extreme reactivity. Conversely, the resilient group may have greater risk for future adverse health outcomes, but also may perform better when they are sleep deprived. Future studies are needed to test these hypotheses.

Our study was conducted in NASA’s HERA mission, which is useful for examining the behavioral and physiological health impacts of various stressors, including sleep deprivation and isolation experienced during spaceflight (Barger et al., 2014; Cromwell et al., 2021; Mhatre et al., 2021); thus, our results demonstrate the importance of considering interindividual differences in vulnerability to sleep loss and stress between astronauts enduring both short and long duration missions. The current results are similar to findings comparing CV measures collected on the ground pre-spaceflight to measures collected post-long-duration spaceflight (Norsk et al., 2015). These parallels are advantageous for better predicting CV changes resulting from long-duration spaceflight, which inevitably includes sleep deprivation and psychosocial stress (Barger et al., 2014; Cromwell et al., 2021; Mhatre et al., 2021). Norsk et al. (2015) found that SV increases with long-duration spaceflight, as was observed in our current experiment, thus demonstrating the potential application of predicting decrements to sleep loss and stress using CV measures in long-duration space missions.

There are a few limitations to this study. All participants in the study were healthy adults. Thus, these data may not be generalizable to clinical populations or to older adults in which lower basal cardiac output has been related to worse cognitive decline (Bown et al., 2020). Our findings may also not be generalizable to scenarios that do not involve isolation; however, isolation is required in studies investigating neurobehavioral and hemodynamic changes in high-fidelity space analogs, such as the present study, in order to simulate space flight conditions. Our results are limited to individual differences during sleep loss on the 10-min PVT and KSS, and our relatively small sample size did not allow for exploration of individual differences in a potential dose-response relationship. Furthermore, we used a median split to classify individuals as resilient or vulnerable or in pre-study low and high groups, which is a commonly utilized method in our field (Chuah et al., 2009; Rocklage et al., 2009; Chee and Tan, 2010; Diekelmann et al., 2010; Patanaik et al., 2015; Yeo et al., 2015; Xu et al., 2016; Moreno-Villanueva et al., 2018; Caldwell et al., 2020; Salfi et al., 2020), although other methods, such as tertile (Chua et al., 2014; Kong et al., 2015) or quartile splits (Chua et al., 2019), have also been utilized. Additionally, VTI of the CW wave form of the ascending aorta was used as a surrogate measure for LVOT VTI. Although echocardiography is a well-validated method to obtain SV, HR, CI, and LVET, using spectral analysis from electrocardiography may obtain a better picture of potential autonomic balance differences between resilient and vulnerable groups. Moreover, the short fasting period prior to biomarker collection may have slightly impacted CV measure outcomes, though this was necessary to maintain consistency throughout the experiment. Lastly, there was a small difference in sleep onset latency between KSS resilient and vulnerable groups on B1, but not on B2; this finding may suggest vulnerable individuals were sleepier on the first, but not the second night of the 5-day experiment, although we did not use a physiological measure of sleepiness (e.g., Maintenance of Wakefulness Test or Multiple Sleep Latency Test) to assess this explicitly.

Our results open the door for research investigating whether pre-study LVET is a biomarker and may identify individual differences in metabolic responses to sleep loss (Spaeth et al., 2015), or be used in conjunction with genetic (Casale and Goel, 2021) and omic studies to further understand the biological factors underlying individual differences in response to TSD and stress (Goel, 2015). We show, for the first time, pre-study LVET differentiates deficits in neurobehavioral performance during TSD and psychosocial stress, may serve as a biomarker, and may inform future health risks.

## Data Availability Statement

The data generated and analyzed during the current study are available from the corresponding author upon reasonable request.

## Ethics Statement

The studies involving human participants were reviewed and approved by the Institutional Review Boards of the NASA and of the University of Pennsylvania. The participants provided their written informed consent to participate in this study.

## Author Contributions

NG designed the overall study and provided financial support. EY conducted statistical analyses of the data. EY, KR-G, CC, LM, AE, JK, and NG prepared the manuscript. All authors reviewed and approved the final manuscript.

## Conflict of Interest

KR-G is currently employed by Siemens Healthineers, a biomedical device manufacturer. KR-G was employed at Wyle Science, Technology and Engineering, Houston, TX, United States during the time when the study was designed, and data were collected. The remaining authors declare that the research was conducted in the absence of any commercial or financial relationships that could be construed as a potential conflict of interest.

## Publisher’s Note

All claims expressed in this article are solely those of the authors and do not necessarily represent those of their affiliated organizations, or those of the publisher, the editors and the reviewers. Any product that may be evaluated in this article, or claim that may be made by its manufacturer, is not guaranteed or endorsed by the publisher.
